# Assessment of blood flow patterns in the pulmonary artery using 4D flow

**DOI:** 10.1186/1532-429X-13-S1-P66

**Published:** 2011-02-02

**Authors:** Pablo Bächler, Natalia Pinochet, Cristián Tejos, Crelier Gerard, Pablo Irrarázabal, Sergio Uribe

**Affiliations:** 1 Pontificia Universidad Católica de Chile, Santiago, Chile; 2 Institute for Biomedical Engineering, University and ETH., Zurich, Switzerland

## Background

**4**D Flow has been used to study flow patterns, mainly in the aorta. In the Pulmonary Artery (PA) an abnormal Vortex (VO) has been described in association with pulmonary hypertension [1]. However, a systematic analysis of flow patterns in the PA has not been performed.

## Objective

To perform hemodinynamic analysis in the PA in volunteers and patients with CHD after Glenn procedure.

## Methods

Eighteen volunteers and two patients underwent 4D Flow scan on a Phillips system (25 frames, 2.5mm^3^). Flow was visualized by streamlines and particle traces ("GTFlow” software). 2D planes placed in 5 locations were used to grade flow patterns: 1) just after to the pulmonary valve, 2) before the PA bifurcation, 3) between plane 1 and 2, 4) Right-PA, and 5) Left-PA. Sagital and coronal planes were also analyzed. VOs that lasted at least 2 frames were registered (start, finish and peak-frame; direction, size, and distance between vortex-center and vessel-center).

## Results

**Figure 1:**
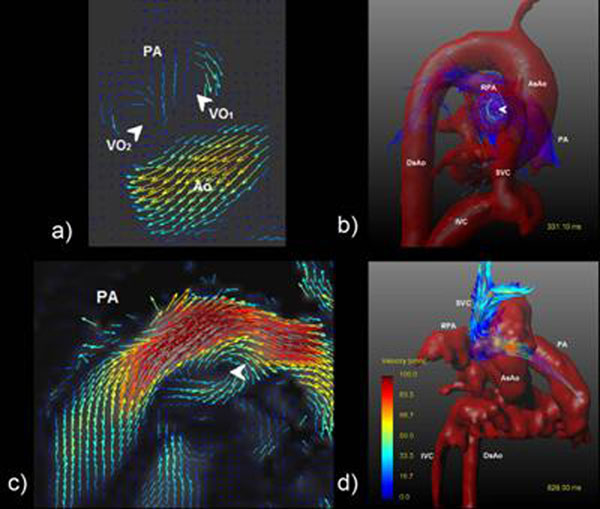
a) and b) Vortices in the PA and Right-PA seen in volunteers. c) Retrograde flow seen in the PA of patients. d)Flow from the SVC to the PA and right ventricle.

Two VOs were identified in the PA in volunteers (Fig. 1a). VO_1_ was seen in all volunteers and VO_2_ in sixteen. Both vortices started at peak-systole and ended at late-systole. VO_1_ was located on posterior wall and VO_2_ on anterior wall, with clockwise and counterclockwise direction respectively. VO_1_ was smaller compared with VO_2_ (24%±7% of vessel area vs. 31%±7%, p-value=0.03). VO_1_’s center was located closer to the vessel’s center compared with VO_2_’s center (10.1mm ± 2mm vs. 11.4mm ± 2mm, p-value=0.02). A clockwise VO was also seen in Right-PA in 15 volunteers (Fig. 1b), starting at peak-systole and ending at early-diastole. VO’s size was on average 51%±20% of Right-PA area.

In both patients abnormal VOs were detected. At systole a VO in PA associated to backward flow was found (Fig. 1c). A main finding was retrograde blood flow from superior vein cava (SVC) at diastole, showing that up to 90% (in one frame) of SVC flow goes toward proximal Right-PA, and more than 70% reaches to the main PA (Fig. 1d).

## Conclusion

VOs are normally founded in PA in healthy subjects. Abnormal blood flow patterns are seen in the PA in patients after Gleen procedure, with most of flow from SVC going to the main PA and right ventricle.
